# What Color is My Arm? Changes in Skin Color of an Embodied Virtual Arm Modulates Pain Threshold

**DOI:** 10.3389/fnhum.2013.00438

**Published:** 2013-07-31

**Authors:** Matteo Martini, D. Perez-Marcos, M. V. Sanchez-Vives

**Affiliations:** ^1^Institut d’Investigacions Biomèdiques August Pi i Sunyer (IDIBAPS), Barcelona, Spain; ^2^EVENT-Lab, Facultat de Psicologia, Universitat de Barcelona, Barcelona, Spain; ^3^Institució Catalana de Recerca i Estudis Avançats (ICREA), Barcelona, Spain

**Keywords:** virtual arm, virtual reality, body ownership, pain threshold, pain modulation, multisensory integration, multisensory stimulation

## Abstract

It has been demonstrated that visual inputs can modulate pain. However, the influence of skin color on pain perception is unknown. Red skin is associated to inflamed, hot and more sensitive skin, while blue is associated to cyanotic, cold skin. We aimed to test whether the color of the skin would alter the heat pain threshold. To this end, we used an immersive virtual environment where we induced embodiment of a virtual arm that was co-located with the real one and seen from a first-person perspective. Virtual reality allowed us to dynamically modify the color of the skin of the virtual arm. In order to test pain threshold, increasing ramps of heat stimulation applied on the participants’ arm were delivered concomitantly with the gradual intensification of different colors on the embodied avatar’s arm. We found that a reddened arm significantly decreased the pain threshold compared with normal and bluish skin. This effect was specific when red was seen on the arm, while seeing red in a spot outside the arm did not decrease pain threshold. These results demonstrate an influence of skin color on pain perception. This top-down modulation of pain through visual input suggests a potential use of embodied virtual bodies for pain therapy.

## Introduction

Color is highly relevant in human visual perception. Colors can affect visual search and attention (Green and Anderson, 1956; Woodman, 2013) and, further, they can affect how a given stimulus is perceived. For example, colors have the power to endorse an implicit meaningful association in relation to temperature. Typically, red is linked to “hot” while blue to “cold” (Moseley and Arntz, 2007). Indeed, a controversial but intuitive hypothesis states that visual appearance of an object (mainly its color) should have some influence on thermal perception (Candas and Dufour, 2005). Durgin and coworkers observed that a blue light beam projected on the hands produces a thermal sensation that was cooler than the one elicited by a red light beam. Furthermore, the same illusory sensation holds true when these lights are pointed to embodied rubber hands (Durgin et al., 2007). Noteworthy is a recent study by Kanaya et al. (2012) who showed how thermal judgments about an object placed on one’s hand are modified according to the thermal property of the object that touches an embodied rubber hand. Nevertheless, whether colors can act as visual modulators of pain perception is still poorly understood. Moseley and Arntz (2007) found that the pain is greater when a stimulus is associated to a red visual cue than when the same stimulus is associated to a blue visual cue. Subsequently, Landgrebe et al. (2008) found that somatosensory perception is altered by colored light exposure, and that diffuse red light decreases cold pain thresholds compared to white and green light, increasing the detection and pain thresholds for warm stimuli. A recent study investigating the effects of the perceived time lapsed during a painful stimulus did not find any influence of the color of the clock on the pain perceived (Peyron et al., 2012).

Different cognitive factors are known to modulate pain perception such as distraction, expectation, emotion, learning, and spatial attention (see Wiech et al., 2008; Legrain et al., 2009 for reviews on the topic). Yet, there is no study up to date on whether the manipulation of the skin color on a body felt as one’s own affects pain perception.

Virtual reality (VR) technology represents a versatile mean for perception studies, as it allows the creation of sensory environments that can be replicated almost identically and that are under the full control of the experimenter (Sanchez-Vives and Slater, 2005). In the present experiment, we investigated whether the vision of different colors applied on an embodied virtual body affected the pain thresholds of healthy volunteers. We tested pain threshold by applying increasing ramps of heat stimuli to the wrist of the subjects while they were concomitantly seeing their virtual arm getting increasingly red, blue, or green. In order to create the illusion of embodiment of the virtual body we used visuo-proprioceptive correlations and first-person perspective with respect to the virtual body. Adequate sensorimotor correlations have been recently proven to be effective in fostering the embodiment of a virtual limb (Slater et al., 2009; Normand et al., 2011; Kilteni et al., 2012; Llobera et al., 2013), including as well as visuo-proprioceptive ones (Kalckert and Ehrsson, 2012). Since the colors blue and red are associated to cold and hot respectively, our hypothesis was that the heat pain threshold would be lower for red than for blue skin color. Further, in order to show that the association color-temperature is maximally effective only when it interests the body, we introduced a condition where the arm was left unaltered and a spot close to the avatar’s arm, but off of it, got increasingly red.

## Materials and Methods

### Participants

Thirty healthy participants (all females, mean ± SD age: 23.9 ± 5.7 years) were recruited for the experiment from the campus of Psychology Sciences of University of Barcelona. They had normal or normal-to-corrected vision, no history of neurological disorders and no other condition potentially interfering with pain sensitivity (e.g., drug intake). Upon arrival at the laboratory they were asked to read and sign a consent form. The experiment was approved by the Comité Ético de Investigación Clínica de la Corporación Sanitaria Hospital Clínic de Barcelona. All participants received a monetary reimbursement for their participation. Importantly, in a debriefing, all subjects could distinguish and correctly identify the colors presented.

### Virtual reality system

The stereoscopic head-mounted display (HMD) was a NVIS SX111 with a resolution of 1280 × 1024 pixels per eye and a total field of view of 111° × 64°, displayed at 60 Hz. The head-tracking was realized with a 6-DOF InterSense IS-900 device (InterSense, Billerica, MA, USA). Finger tracking was permitted by attaching two markers on a plastic ring put on the participant’s finger. These markers were constantly tracked by 12 infrared OptiTrack cameras, and their coordinates in the space computed with the Arena software (NaturalPoint, Corvallis, OR, USA). Hence, when the participant’s finger was moved, the avatar’s finger could move accordingly, mimicking exactly the same movements at the same time. The virtual environment was programed using the XVR system (Tecchia et al., 2010) and the virtual body using the HALCA library (Gillies and Spanlang, 2010). Noise isolation was ensured by the administration of pink noise through a surround audio system (Creative technology Ltd., Singapore), with a constant volume set at 65 dB SPL.

### Thermal stimulation

Thermal heat stimuli were delivered by means of a Thermotest machine (Somedic, Hörby, Sweden) with a 2.5 cm × 5.0 cm thermode tied with a Velcro strap on the palmar side of the right wrist. Pain thresholds were assessed with the method of limits (Yarnitsky et al., 1995). The probe temperature was increased from normal skin temperature (constant baseline temperature = 31°C) at 2°C/s. Participants were asked to press a button with their left hand as soon as they perceived the stimulation as being painful. Immediately after pushing the kill-switch button, the probe temperature rapidly decreased to the baseline temperature. For safety reasons, maximal temperature was set at 48°C.

### Procedure

**Figure 1:**
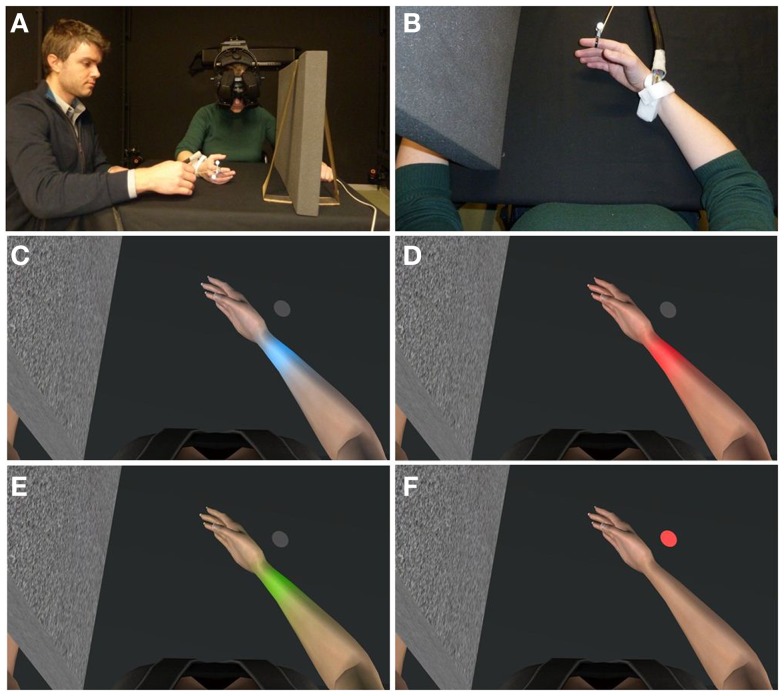
**The experimental set-up and the four experimental conditions: the participant saw the virtual environment through the HMD while an experimenter moved her right index finger to move the avatar’s right index finger accordingly**. When the heat stimulation provided on the right wrist was felt as painful by the participant, she stopped the stimulation by pressing the button held on her left hand **(A)**. Top view of the posture of participant’s arm resting on the table, matching avatar’s posture **(B)**. The visuo-proprioceptive congruent feedback given by the finger movements and the first-person perspective view of the avatar fostered the embodiment of the virtual limb while the skin color changed into blue **(C)**, red **(D)**, or green **(E)** as soon as the heat stimulation started increasing in temperature. In the fourth condition, the skin of the virtual arm did not change color but a gray spot on the table turned into red **(F)**.

Participants sat on a chair with both arms resting on a table covered with a black cloth (Figures 1A,B). Before donning the HMD, they were given two to three heat stimuli to familiarize with the heat ramps.

As the subject donned the HMD the room’s lights were turned off and the pink noise played. The HMD allowed participants to experience an immersive virtual environment around them and to see a virtual body, from a first-person perspective, in place of their own (Kilteni et al., 2012). Additionally, they were asked to move their right arm where they saw that the avatar’s arm was, and keep it in the same position so that, when participants looked down at their own body, they could see the virtual body perfectly co-located with their own. They were also asked to place the hand and the arm mimicking as much as possible the position of the virtual ones. The left virtual arm was hidden behind a virtual foam shield, in order for the subject to see and concentrate only on the right arm. Importantly, both the virtual right hand and forearm were always kept in the field of view of the participants. Precisely, in all conditions, they were asked to focus their attention on the wrist only, while the finger movements clearly remained in their field of view. As the experiment started, an experimenter moved the participant’s right index finger continuously in an outward-inward fashion. This passive movement was meant to provide the proprioceptive feedback without calling into play motor control and thus the role of agency (Kalckert and Ehrsson, 2012), and to ensure that all subjects constantly saw the finger moving throughout the whole experiment. Further, the correspondence between the visual and the proprioceptive inputs provided the experience of “embodying” the avatar’s limb, a phenomenon already documented as “virtual hand illusion” (Slater et al., 2008, 2009). Four different visual conditions were presented to all participants (Figure 1), with the avatar’s wrist becoming either blue (Figure 1C), red (Figure 1D), or green (Figure 1E). In a different condition, a gray spot placed on the virtual table, close to the participant’s wrist, became red (Figure 1F). Each color transformation was presented four times for a total of 16 visual stimuli. In order to limit habituation to the same visual experience, stimuli were presented in an event-related fashion (intermixed). In each trial, the increase of the thermal stimulation was concomitant to the change of color (toward red, green, or blue) of the avatar’s wrist and nearby skin area. In the “spot” condition, the increase of the stimulus temperature coincided with the round spot becoming red, while there was no change of the avatar’s skin color. The color change started at the same time than the thermal stimulation and lasted for 3 s in both directions (color appearance and disappearance), independently of the condition. The color intensity increased/decreased linearly and reached its maximum when the temperature was 37°C, i.e., before reaching the pain threshold range (starting around 40°C). The color started disappearing once the participant had stopped the thermal stimulation. The order of the visual stimuli was pseudo-randomized across subjects, in order to avoid that one visual condition was affected more than others by habituation to the painful stimulation (Greffrath et al., 2007). The inter-stimulus interval was set at a random pace between 45 and 60 s. A pause of 2 min was introduced after the 8th stimulus to prevent from possible neck and head muscles fatigue (caused by the weight of the HMD and the inclination of the head).

### Subjective measures

Subjective report about the level of embodiment was collected on a single trial basis immediately after each thermal stimulation. Subjects were instructed to spell out a number in order to reply to the question: “by the time you were seeing the color appearing on the wrist, did you feel as if the virtual right arm was your own right arm?”. This item was referred as to the “embodiment level” and measured with a seven points Likert scale. A score of “1” meant “not at all” while “7” stood for “yes, completely.”

### Data handling

Pain thresholds (in °C) were averaged per each one of the visual conditions and subject. These were normally distributed according to the Kolmogorov–Smirnov test (*p* > 0.05). Five (out of 120) scores from two subjects were identified as outliers (higher than twice the standard deviation from the group’s mean) and replaced with the mean scores of the group for the same visual condition (i.e., if the score was related to the “green arm” condition, it was replaced with the mean score obtained for the “green arm” condition). One-way repeated-measures ANOVA (one factor: “Condition” with four levels) was then conducted on mean pain thresholds. *Post hoc* analysis was conducted with Tukey HSD tests. The significance level was set at *p* < 0.05.

The scores reported for the “embodiment level” were averaged per each visual condition and participant, and then subjected to a Friedman ANOVA. *Post hoc* analysis with Wilcoxon Matched Pairs Tests was conducted, with a Bonferroni correction applied for the number of possible comparisons. This resulted in a significance level set at *p* < 0.008. Statistical comparisons between conditions were conducted with STATISTICA (StatSoft, Inc., Tulsa, OK, USA).

## Results

### Pain threshold

**Figure 2:**
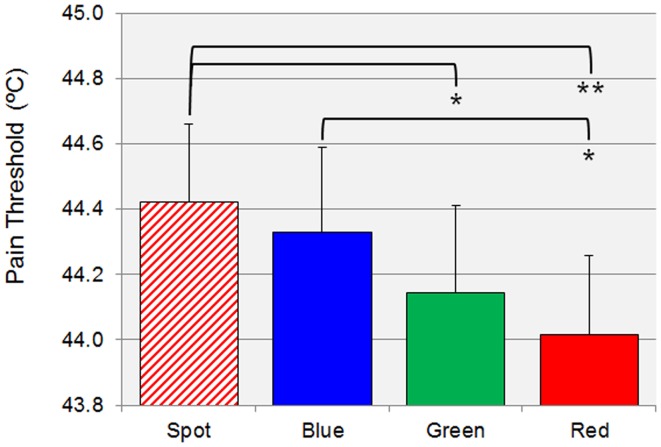
**Columns and vertical error bars respectively stand for group means and standard errors of the pain thresholds in each condition**. Asterisks indicate significant comparisons (*p < 0.05; **p < 0.01).

The one-way repeated-measures ANOVA showed an effect of the factor “Condition” (*F*_3, 87_ = 5.93, *p* < 0.001), meaning that the vision of different colors influenced pain thresholds (Figure 2). Tukey *post hoc* tests revealed that the vision of the blue arm led to a higher pain threshold compared to the one reported in the red arm condition (*p* = 0.020).

A significantly higher pain threshold was also detected while participants were seeing the red spot on the table compared either to the red arm (*p* = 0.001) and the green arm (*p* = 0.046). No other comparison was found to be significant.

### Embodiment scores

The analysis with Friedman ANOVAs on the embodiment scores reported a significant *p-*level ($\chi_{3}^{2}=9.09,$ *p* = 0.028). The embodiment level reported in the “red arm” condition was higher than that obtained in the “blue arm” (*p* = 0.045). However, *post hoc* comparisons did not confirm the statistical significance (Wilcoxon *post hoc* test, *p*-corrected level = 0.008). This means that there was no actual difference in terms of embodiment between conditions. No other comparison was found to be significant.

## Discussion

Mean ratings relative to embodiment level are displayed in Table 1. The present experiment tested for the first time whether the vision of different colors presented on the body affected heat pain perception. By means of immersive VR participants internalized a virtual body, thus permitting experimentally controlled visual changes of it. Our results evidence that the vision of different skin colors on the embodied virtual body affects pain threshold. Specifically, when subjects saw the virtual limb becoming blue there was a significant increase of pain threshold compared to when they saw it getting red. Moreover, we also show that pain threshold is affected differently depending on whether color is presented on or off the body. An important finding is that the vision of the red color *per se* was not always associated to a decrease of the pain threshold. Rather, when a cue close to the avatar’s arm, but off of it, got red, we registered the highest pain thresholds, significantly greater than the one recorded with the arm becoming either red or green.

**Table 1:** **Means (± SD) of the ratings relative to the embodiment question per each visual condition**.

Condition	Embodiment ratings
Blue	5.47 ± 1.09
Red	5.75 ± 0.85
Green	5.48 ± 0.99
Red spot	5.67 ± 0.97

Following the “hue-heat hypothesis,” which states that colors toward the red end of the visual spectrum are perceived as “warm” and those toward the blue end as “cool,” the idea that the vision of a particular color can be associated to a specific temperature is gaining experimental evidences (Landgrebe et al., 2008; Michael et al., 2010; Kanaya et al., 2012). Moseley and Arntz (2007) showed that when the noxious stimulus is associated with a red visual cue, it hurts more and it is actually perceived as hotter than when the same stimulus is associated with a blue visual cue signaling that the stimulus is “cold,” although actually it is not. In a following investigation, fourteen healthy volunteers with normal color vision assessed temperature perception during exposure to different lights: colored lights exposure did alter somatosensory perception, with red light decreasing cold pain thresholds compared to white light, and green light increasing the detection and pain thresholds for warm stimuli (Landgrebe et al., 2008). One recent study, however, failed to find any specific regulatory effect on pain by the vision of a green or a red clock (Peyron et al., 2012). Our results uphold the idea that temperature-related colors, i.e., red and blue, may affect heat pain threshold and this effect changes according to whether they are seen on one’s body or out of it. How can the vision of red and blue do that? Likely, the meaning attributed to the colored cue plays a major role in determining pain perception. For example, it could be that the vision of an intense reddened skin would bring the meaning that the arm is getting harmed by heat, conveying a threatening message and thus leading to an increase of the experience of pain (Arntz and Claassens, 2004). Seeing a blue arm instead would mean that a “cooling” of the skin is taking place, thus contrasting with the heating of the thermode and so yielding a higher pain threshold. In fact, when the body is exposed to different temperatures it reacts with thermoregulation through superficial vasodilation when it’s hot, or vasoconstriction when it’s cold; this in turn brings the tissues to be highly or poorly oxygenated by hemoglobin, which renders the skin red or blue (cyanotic), respectively (Hirschmann and Raugi, 2009; Everett et al., 2012).

Why the vision of a reddening cue out of the body led to the highest pain threshold? It is known that the experience of pain is modified by the direction of spatial attention, so that directing attention away from the pain location results in a reduction of pain. For instance, in a recent study participants perceived painful stimuli as significantly less painful when visual cues were presented at a different location from where the painful stimuli were applied, in comparison with the condition where visual cues were presented at the same location of the painful stimuli (Ryckeghem et al., 2011). Hence, in the present experiment, having a visual cue on the virtual table may have directed a shift of the spatial attention toward the cue as it started changing color (from gray to red), despite the instructions to focus only on the virtual wrist. Yet, the introduction of a technique allowing the measurement of the gaze direction of the participants (e.g., by means of eye tracking) would have provided an empirical support to this claim.

As expected, because the green color is usually not related to any temperature directly, it did not drive the pain threshold into a clear direction. Indeed, our data show that pain threshold during the vision of the arm getting green is between the bluish and the reddened arms (higher and lower, respectively). Most participants anecdotally reported a connection between a blue arm with a cold, and red arm with a warm sensation. However, only one reported a connection between the vision of green and a temperature-related ideation. This would rule out the possibility that the green color was generally associated to any temperature. So, given that the green color is temperature-unrelated (at least not related as red and blue are) and that the pain threshold value reported with the vision of the green arm stood just between the red and the blue arm, we could say that a bluish arm augments the heat pain threshold while a reddish one decreases it. Landgrebe et al. (2008) have shown that the vision of a green light would increase the heat pain threshold as compared to the vision of either a white or red light. Yet, we did not find any significant difference in pain threshold between the green and red conditions. This apparent incongruence could be due to several important differences between our study and Landgrebe’s one. The following differential factors may explain these divergences: (i) the lights used in Landgrebe experiments were diffuse/environmental lights while, in our study, colors specifically implied a change of the embodied limb (except for the “spot” condition); (ii) the sample size of the present study (*n* = 30) doubled that from Landgrebe’s study, which is relatively small (*n* = 14); and (iii) their sample was composed of male subjects only while ours was formed by female subjects only. As suggested by the authors, “gender might influence the cross-modal effects of colour” (Landgrebe et al., 2008).

Recently, it has been reported that the illusion of owning a rubber hand does not induce any significant change in the perception of pain (Mohan et al., 2012). Nevertheless, the focus of the present study was not to compare the effects of embodiment on pain threshold itself but, rather, to see whether the color of the skin of an embodied virtual arm could affect the pain threshold. Our results suggest a strong relationship between the vision of the skin color and the expected temperature, which may exert a top-down modulation of the pain threshold. This may reveal fundamental implications for the design of multimodal therapy approaches for the treatment of pain states that include visual feedback of the own body or of an embodied avatar.

## Conflict of Interest Statement

The authors declare that the research was conducted in the absence of any commercial or financial relationships that could be construed as a potential conflict of interest.
